# Perioperative hemodynamic instability in pheochromocytoma and sympathetic paraganglioma patients

**DOI:** 10.1038/s41598-021-97964-3

**Published:** 2021-09-17

**Authors:** Jung Hee Kim, Hyung-Chul Lee, Su-jin Kim, Soo Bin Yoon, Sung Hye Kong, Hyeong Won Yu, Young Jun Chai, June Young Choi, Kyu Eun Lee, Kwang-Woong Lee, Seung-Kee Min, Chan Soo Shin, Kyu Joo Park

**Affiliations:** 1 Department of Internal Medicine, Seoul National University College of Medicine, Seoul National University Hospital, Seoul, Republic of Korea; 2 Department of Anesthesiology and Pain Medicine, Seoul National University College of Medicine, Seoul National University Hospital, Seoul, Republic of Korea; 3 Department of Surgery, Seoul National University College of Medicine, Seoul National University Hospital, Seoul, Republic of Korea; 4 Cancer Research Institute, Seoul National University College of Medicine, Seoul, Republic of Korea; 5 Division of Surgery, Thyroid Center, Seoul National University Cancer Hospital, Seoul, Republic of Korea; 6 Medical Big Data Research Center, Institute of Medical and Biological Engineering, Seoul National University, Seoul, Republic of Korea; 7 Department of Surgery, Seoul National University Bundang Hospital, Seongnam, Republic of Korea; 8 Department of Surgery, Seoul National University Boramae Medical Center, Seoul, Republic of Korea

**Keywords:** Cancer, Endocrinology, Medical research

## Abstract

For pheochromocytoma and sympathetic paraganglioma (PPGL), surgery can be used as a curative treatment; however, the life-threatening risk of perioperative hemodynamic instability (HI) presents challenges. This study aimed to analyze the incidence and predictive factors of perioperative HI. The electronic medical records of 114 consecutive patients who underwent surgery for PPGLs at our institution were retrospectively reviewed. HI was defined as one or more episodes of systolic blood pressure > 200 mmHg or mean blood pressure < 60 mmHg during surgery. The factors predictive of perioperative HI were determined using both univariate and multivariate analyses. Intraoperative HI occurred in 79 (69.3%) patients. In multivariate analysis, α-adrenergic receptor blocker duration (days) (odds ratio, 1.015; 95% confidence interval, 1.001–1.029) was a predictor for intraoperative HI. Postoperative hypotension occurred in 36 (31.6%) patients. Higher urine epinephrine levels, and greater preoperative highest heart rate (HR) were predictive factors for postoperative hypotension in PPGL patients. Caution should be taken in perioperative management for PPGL, especially with long duration of α-adrenergic receptor blocker use, higher urine epinephrine levels, and greater preoperative highest HR.

## Introduction

Pheochromocytoma and sympathetic paraganglioma (PPGL) are rare neuroendocrine tumors arising from catecholamine-producing chromaffin cells in the adrenal medulla and extraadrenal sympathetic paraganglia. The overall prevalence of PPGL was 2.13 per 100,000 persons, and the overall age-standardized incidence rate was 0.18 per 100,000 person years^1^. Excessive catecholamine release from PPGLs can induce life-threatening cardiovascular manifestations, including hypertensive crisis, myocardial infarction, brady- and tachyarrhythmias, Takotsubo cardiomyopathy, and acute heart failure^2,3^.

Surgery can be used as a curative treatment for these tumors to prevent cardiovascular and other organ system complications associated with catecholamine excess^4^. However, surgery is considered challenging due to the life-threatening risk of perioperative hemodynamic instability (HI). Perioperative HI, characterized by arrhythmias, abrupt increases in blood pressure (BP) during manipulation of the tumor, and decreases in BP after ligation of the adrenal vein can be major challenges for perioperative management of PPGL patients^5,6^.

To avoid HI, routine use of α blockade and volume expansion has been incorporated into the perioperative optimization of the patient’s condition before surgery^4,7^. Despite proper preoperative preparation for PPGL, HI is unpredictable and poorly defined. Recent studies have analyzed the predictive factors for HI, but the results are conflicting^8–10^. There are several potential predictive factors for HI: a large tumor size^9,10^, the type of operation (open vs. laparoscopic)^10^, the type of α blockade^10^, symptomatic preoperative high BP^8^, and high levels of catecholamine^8^. In addition, the incidence of HI is highly variable in previous studies, such as in reports by Gajoux et al.^8^ (8.7%, 13/149) and Jiang et al.^9^ (67%, 44/134). This discrepancy is thought to be due to various definitions of HI in these studies, the lack of any prospective studies reducing the selection bias, and the small study populations.

The aim of this study was to evaluate the incidence of HI during surgery and to analyze the predictive factors for intraoperative HI and postoperative hypotension in PPGL patients.

## Results

Among 114 subjects, HI occurred in 79 (69.3%) subjects. There are 10 (12.7%, 10/79) patients with SBP > 200 mmHg, and 76 (96.2%, 76/79) patients with MBP < 60 mmHg. The clinical and biochemical characteristics of the study subjects, according to HI, are compared in Tables 1 and 2. The demographic, clinical, and biochemical variables were similar in subjects with and without HI. The type of α-adrenergic receptor blocker was not different between the two groups. However, the duration of α-adrenergic receptor blocker use was longer in subjects with HI than in subjects without HI (63.8 ± 57.3 vs. 40.7 ± 33.0 days, P = 0.008) (Supplemental Fig. 1). American Society of Anesthesiologists (ASA) class, surgical approach, anesthesia, and duration of surgery were not different between the two groups.

**Table 1:** Clinical characteristics of study subjects according to hemodynamic instability (HI).

Variables	HI (−)	HI (+)	Total	*P*-value
	(N = 35)	(N = 79)	(N = 114)	
Age (years)	49.1 ± 14.4	49.9 ± 16.0	49.6 ± 15.5	0.800
Female	16 (45.7%)	39 (49.4%)	55 (48.2%)	0.875
Body weight (kg)	63.9 ± 11.4	62.9 ± 11.3	63.2 ± 11.3	0.671
BMI (kg/m^2^)	23.6 ± 3.3	23.7 ± 3.8	23.6 ± 3.7	0.764
Diabetes mellitus	6 (17.1%)	22 (27.8%)	28 (24.6%)	0.323
Hypertension	17 (48.6%)	43 (54.4%)	60 (52.6%)	0.708
Coronary artery disease	7 (20.0%)	9 (11.4%)	16 (14.0%)	0.249
Cerebrovascular disease	3 (8.6%)	6 (7.6%)	9 (7.9%)	1.000
History of abdominal surgery	5 (14.3%)	17 (21.5%)	22 (19.3%)	0.519
Symptomatic presentation	18 (51.4%)	43 (54.4%)	61 (53.5%)	0.926
Maximal size (cm)	4.3 ± 2.1	5.1 ± 2.8	4.8 ± 2.6	0.212
**Side**				0.194
Right	7 (25.9%)	29 (44.6%)	36 (39.1%)	
Left	17 (63.0%)	28 (43.1%)	45 (48.9%)	
Bilateral	3 (11.1%)	8 (12.3%)	11 (12.0%)	
Metastasis	1 (2.9%)	7 (8.9%)	8 (7.0%)	0.606
Multifocal	1 (2.9%)	5 (6.3%)	6 (5.3%)	0.326
**Preoperative vital signs**				
Preblockade SBP (mmHg)	134.1 ± 15.1	129.2 ± 20.7	130.7 ± 19.2	0.196
Lowest SBP (mmHg)	106.2 ± 10.9	101.9 ± 10.7	103.3 ± 10.9	0.058
Highest SBP (mmHg)	150.4 ± 23.1	144.2 ± 24.4	146.2 ± 24.1	0.118
Lowest HR (bpm)	60.0 ± 10.9	62.3 ± 12.5	61.6 ± 12.0	0.231
Highest HR (bpm)	91.4 ± 18.0	93.5 ± 19.5	92.9 ± 19.0	0.866
**ASA**				0.351
1	5 (14.3%)	10 (13.2%)	15 (13.5%)	
2	20 (57.1%)	52 (68.4%)	72 (64.9%)	
3	9 (25.7%)	14 (18.4%)	23 (20.7%)	
4	1 (2.9%)	0 (0.0%)	1 (0.9%)	
**Surgical approach**				0.896
Open*	13 (37.1%)	32 (40.5%)	45 (39.5%)	
Laparoscopic	22 (62.9%)	47 (59.5%)	69 (60.5%)	
Duration of anesthesia (min)	178 (157–224)	193 (147–252)	185 (148–247)	0.398
Duration of surgery (min)	110 (83–172)	125 (80–185)	123 (80–185)	0.341

Data are presented as the mean ± standard deviation, median (interquartile range) or n (%). HI, hemodynamic instability; SBP, systolic blood pressure; ASA, American Society of Anesthesiologists; *12 cases of open conversion were included in the open approach.

**Table 2:** Biochemical characteristics, and medications of study subjects according to hemodynamic instability (HI).

Variables	HI (−)	HI (+)	Total	*P*-value
	(N = 35)	(N = 79)	(N = 114)	
**Hormone type**				0.076
Nonfunctioning	5 (14.3%)	2 (2.5%)	7 (6.1%)	
Epinephrine	19 (54.3%)	50 (63.3%)	69 (60.5%)	
Norepinephrine	11 (31.4%)	27 (34.2%)	38 (33.3%)	
Serum metanephrines (pg/mL)	2350.0 ± 3116.0	5593.5 ± 7226.2	4603.4 ± 6423.0	0.089
Serum normetanephrines (pg/mL)	8486.2 ± 7492.1	8647.4 ± 7363.5	8597.1 ± 7363.4	0.930
Urine epinephrine (µg/day)	45.0 ± 88.1	126.2 ± 376.4	102.1 ± 320.7	0.147
Urine norepinephrine (µg/day)	458.3 ± 512.8	500.4 ± 799.9	487.9 ± 724.4	0.824
Urine dopamine (µg/day)	247.3 ± 79.2	342.7 ± 310.7	314.4 ± 267.0	0.223
Urine metanephrines (µg/day)	912.4 ± 1597.2	2275.8 ± 5972.0	1870.5 ± 5108.7	0.197
Urine normetanephrines (µg/day)	3049.0 ± 3212.6	5110.5 ± 12,925.3	4497.6 ± 10,992.9	0.854
**Medications**				
α-blocker use	32 (91.4%)	73 (92.4%)	105 (92.1%)	1.000
α-blocker use				
Phenoxybenzamine	9 (28.1%)	16 (21.9%)	25 (23.8%)	1.000
Doxazosin	23 (65.7%)	49 (62.0%)	72 (63.2%)	
Terazosin	0 (0%)	8 (0.10%)	8 (7.0%)	
Duration of α-blocker use (days)	40.7 ± 33.0	63.8 ± 57.3	56.7 ± 52.0	**0.008**
α-blocker dose (DDD)	0.8 ± 0.5	0.9 ± 0.6	0.8 ± 0.6	0.576
β-blocker use	22 (62.9%)	45 (57.0%)	67 (58.8%)	0.701
Duration of β-blocker use (days)	15.6 ± 55.7	27.8 ± 80.9	67.3 ± 31.8	0.183
β-blocker dose (DDD)	0.3 ± 0.6	0.3 ± 0.8	0.3 ± 0.7	0.257
Calcium channel blocker (DDD)	0.5 ± 0.6	0.4 ± 0.6	0.4 ± 0.6	0.393
RAS inhibitor (DDD)	0.3 ± 0.5	0.5 ± 0.9	0.4 ± 0.8	0.527
Anti-HTN drug (DDD)	1.3 (0.9–2.3)	1.6 (0.8–2.7)	1.3 (0.9–2.7)	0.725

Data are presented as the mean ± standard deviation, median (interquartile range) or n (%). HI, hemodynamic instability; SBP, systolic blood pressure; DDD, daily defined dose; HTN, hypertension.

We conducted logistic regression models of preoperative risk factors for HI (Table 3). Among the variables shown in Tables 1 and 2, the variables with a *P*-value less than 0.1, including the duration of α-adrenergic receptor blocker use, serum metanephrine level, urine dopamine level, and lowest preoperative systolic blood pressure (SBP), were included in the logistic regression models. In the multivariate analysis, the duration of α-adrenergic receptor blocker use (odds ratio [OR], 1.015; 95% confidence interval [CI], 1.001–1.029) was a predictive factor for intraoperative HI.

**Table 3:** Logistic regression analysis for predicting intraoperative hemodynamic instability in PPGL patients. Variables with a *P*-value < 0.1 in univariate analysis were included in the multivariate logistic regression models.

Variables	Univariate		Multivariate	
	OR	*P*-value	OR	*P*-value
α-blocker duration	1.012 (1.001–1.023)	0.033	**1.015 (1.001–1.029)**	**0.041**
Serum metanephrines (pg/mL)	1.0004 (1.0001–1.0007)	0.036	1.000 (1.000–1.000)	0.060
Urine dopamine (µg/day)	1.004 (1.000–1.008)	0.075		
Preoperative lowest SBP	0.964 (0.928–1.002)	0.062		

Data are shown as odds ratios (ORs) (95% confidence intervals). PPGL, pheochromocytoma and sympathetic paraganglioma.

Table 4 demonstrates the intraoperative characteristics of the study subjects according to the HI. Subjects with HI exhibited lower mean SBP and mean blood pressure (MBP) but a longer time when SBP was higher than 200 mmHg and a longer time when MBP was less than 60 mmHg than those without HI. RBC transfusion and the administered doses of phenylephrine and norepinephrine were greater in subjects with HI than in those without HI. However, the dose of vasodilators was not different between the two groups.

**Table 4:** Intraoperative characteristics of the study subjects.

	HI (−) (n = 35)	HI (+) (n = 79)	Total (N = 114)	*P*-value
**Hemodynamic variables**				
Mean SBP	119.3 ± 8.9	112.9 ± 10.6	114.8 ± 10.5	< 0.001
Maximum SBP	188 (169–206)	190 (164–211)	189 (164.5–209.5)	0.847
Minimum SBP	84 (74–90)	68 (55–76)	72 (62–80)	< 0.001
Mean MBP	86.9 ± 6.1	79.7 ± 6.1	81.9 ± 7.0	< 0.001
Maximum MBP	138 (124–148)	132 (118–160)	134 (120–151.5)	0.511
Minimum MBP	60 (57–67)	48 (43–52)	50 (46–58)	< 0.001
Mean HR	69.4 ± 11.3	70.1 ± 13.8	69.9 ± 13.0	0.770
Maximum HR	102.1 ± 16.1	104.6 ± 21	103.8 ± 19.5	0.490
Minimum HR	49.3 ± 9	50.1 ± 11.4	49.8 ± 10.7	0.706
**Hemodynamic instabilities**				
Time SBP > 200 (min)	0.0 ± 0.0	0.9 ± 3.2	0.6 ± 2.7	0.028
Time MBP < 60 (min)	0.0 ± 0.0	16.8 ± 16.2	11.7 ± 15.6	< 0.001
Area SBP > 200 (mmHg × min)	0.0 ± 0.0	24.3 ± 105.4	16.8 ± 88.3	0.028
Area MBP < 60 (mmHg × min)	0.0 ± 0.0	97.1 ± 121.7	67.3 ± 110.6	< 0.001
Std of SBP	19.4 ± 5.1	22.5 ± 5.5	21.5 ± 5.6	0.004
HIS	20.0 (14.0–28.5)	30.0 (22.0–44.0)	28.0 (19.2–36.8)	< 0.001
ARV	15.7 (10.7–19.4)	16.4 (12.7–20.4)	16.2 (12.1–20.0)	0.260
MDAPE	13.1 (9.0–16.5)	15.7 (12.8–20.5)	14.6 (11.5–18.9)	0.002
**Fluid management**				
Urine output (mL)	300 (125–350)	205 (95–458)	240 (100–400)	0.773
Estimated blood loss (mL)	100 (0–325)	100 (25–425)	100 (0–350)	0.386
Total infused fluid (mL)	1350 (725–1750)	1400 (825–2250)	1375 (800–2000)	0.163
Tidal transfused RBC (pack)	0.0 (0.0–0.0)	0.0 (0.0–400.0)	0.0 (0.0–0.0)	0.030
**Vasopressors**				
Use of vasopressor	18 (75.0%)	53 (84.1%)	71 (81.6%)	0.361
Number of vasopressors	1.3 ± 0.9	1.8 ± 1.3	1.7 ± 1.2	0.120
Phenylephrine (µg)	30 (0–105)	100 (15–325)	60 (0–200)	0.028
Norepinephrine (µg)	0.0 (0.0–37.4)	0.0 (0.0–0.0)	0.0 (0.0–60.3)	0.014
Epinephrine (µg)	0.0 (0.0–0.0)	0.0 (0.0–0.0)	0.0 (0.0–0.0)	0.177
Ephedrine (mg)	5.0 (0.0–11.2)	10.0 (0.0–20.0)	5.0 (0.0,15.0)	0.295
Vasopressin (U)	0.0 (0.0–0.0)	0.0 (0.0–0.0)	0.0 (0.0–0.0)	0.245
**Vasodilators**				
Use of vasodilator	15 (62.5%)	46 (73.0%)	61 (70.1%)	0.487
Number of vasodilators	1.1 ± 1.1	1.2 ± 1.1	1.2 ± 1.1	0.670

Data are presented as the mean ± standard deviation or median (interquartile range).

HI, hemodynamic instability; RBC, red blood cell; SBP, systolic blood pressure; MBP, mean blood pressure; Std, standard deviation; HIS, hemodynamic instability score; ARV, average real variability; MDAPE, median absolute performance error.

Supplemental Table 1 demonstrates intraoperative HI according to phenoxybenzamine use. Phenoxybenzamine was not superior to other α-blockers, such as doxazosin or prazosin.

Postoperative hypotension (one or more episodes of MBP < 60 mmHg) occurred in 36 (31.6%) subjects. We further analyzed the clinical predictors for postoperative hypotension in PPGL subjects. Female sex, lower BMI, symptoms, higher normetanephrine and norepinephrine levels, greater highest heart rates (HR), and higher antihypertensive medication dose were related to postoperative hypotension (Table 5). In the multivariate analysis, subjects with higher urine epinephrine levels (OR, 1.003; 95% CI, 1.000–1.007) and greater preoperative highest HR (OR, 1.046; 95% CI, 1.014–1.080) had a higher risk of postoperative hypotension (Table 6). There was two readmissions due to pleural effusion, and one readmission due to ileus. There was no mortality within 30 days of surgery among the subjects.

**Table 5:** Clinical predictors for postoperative hypotension in PPGL patients.

Variables	Postoperative hypotension (−)	Postoperative hypotension (+)	Total	*P*-value
	(N = 78)	(N = 36)	(N = 114)	
Age (years)	50.2 ± 14.9	48.4 ± 16.8	49.6 ± 15.5	0.582
Female	32 (41.0%)	23 (63.9%)	55 (48.2%)	**0.039**
Body weight (kg)	65.0 (57.3–71.0)	58.6 (52.0–64.0)	62.9 (55.0–69.4)	**0.004**
BMI (kg/m^2^)	24.0 (21.8–26.0)	22.4 (20.8–23.7)	23.1 (21.2–25.4)	**0.041**
Symptomatic presentation	36 (46.2%)	25 (69.4%)	61 (53.5%)	**0.034**
Maximal size (cm)	3.8 (2.6–5.0)	5.5 (3.5–6.7)	4.0 (3.0–5.5)	**0.005**
Metastasis	4 (5.1%)	4 (11.1%)	8 (7.0%)	0.259
Multifocal	2 (2.6%)	4 (11.1%)	6 (5.3%)	0.078
**Hormone type**				
Nonfunctioning	6 (7.7%)	1 (2.8%)	7 (6.1%)	0.492
Epinephrine	45 (57.7%)	24 (66.7%)	69 (60.5%)	
Norepinephrine	27 (34.6%)	11 (30.6%)	38 (33.3%)	
Serum metanephrines (pg/mL)	3949.1 ± 5376.3	6168.9 ± 8328.8	4603.4 ± 6423.0	0.201
Serum normetanephrines (pg/mL)	**7233.4 ± 7057.0**	**11,930.7 ± 7147.6**	**8597.1 ± 7363.4**	**0.006**
Urine epinephrine (µg/day)	51.7 ± 99.4	207.0 ± 534.3	102.0 ± 320.7	0.092
Urine norepinephrine (µg/day)	**323.6 ± 456.7**	**830.1 ± 1014.8**	**487.9 ± 724.4**	**0.007**
Urine dopamine (µg/day)	290.0 ± 219.5	365.0 ± 344.1	314.3 ± 267.1	0.238
Urine metanephrines (µg/day)	1198.8 ± 1983.5	3269.8 ± 8410.0	1870.5 ± 5108.8	0.153
Urine normetanephrines (µg/day)	4398.7 ± 12,976.8	4703.6 ± 4866.8	4497.6 ± 10,992.9	0.858
**Preoperative vital signs**				
Preblockade SBP (mmHg)	129.7 ± 19.8	133.7 ± 17.4	130.7 ± 19.2	0.375
Lowest SBP (mmHg)	104.6 ± 10.7	100.5 ± 10.9	103.3 ± 10.9	0.073
Highest SBP (mmHg)	146.8 ± 23.4	145.0 ± 25.7	146.2 ± 24.1	0.730
Lowest HR (bpm)	60.7 ± 11.8	63.5 ± 12.4	61.6 ± 12.0	0.273
**Highest HR (bpm)**	**89.1 ± 15.7**	**100.8 ± 22.7**	**92.9 ± 19.0**	**0.008**
**Medications**				
α-blocker use	70 (89.7%)	35 (97.2%)	105 (92.1%)	0.268
α-blocker type				0.244
Phenoxybenzamine	20 (25.6%)	5 (13.9%)	25 (21.9%)	
Others	58 (74.4%)	31 (86.1%)	89 (78.1%)	
Duration of α-blocker use	44.5 (25.2–80.0)	42.0 (19.0–65.2)	44.0 (22.2–75.5)	0.567
α-blocker dose (DDD)	0.7 (0.5–1.0)	0.9 (0.6–1.1)	0.8 (0.5–1.0)	0.108
β-blocker use	46 (59.0%)	21 (58.3%)	67 (58.8%)	0.889
Duration of β-blocker use	2.0 (0.0–19.8)	2.5 (0.0–14.2)	2.0 (0.0–19.8)	0.982
β-blocker dose (DDD)	0.1 (0.0–0.3)	0.1 (0.0–0.5)	0.1 (0.0–0.3)	0.366
**Anti-HTN drug (DDD)**	**1.1 (0.6–2.3)**	**1.9 (1.2–3.4)**	**1.3 (0.9–2.7)**	**0.029**

**Table 6:** Logistic regression analysis for predicting postoperative hypotension in PPGL patients. Variables with a *P*-value < 0.05 in univariate analysis were included in the multivariate logistic regression models, and forward stepwise selection based on the Wald test was performed.

Variables	Univariate		Multivariate	
	OR	*P*-value	OR	*P*-value
Female sex	2.543 (1.124–5.752)	0.025		
Maximal size (cm)	1.194 (1.013–1.408)	0.035		
Symptomatic presentation	2.652 (1.148–6.125)	0.022		
Multifocal	5.516 (1.096–27.774)	0.038		
Urine epinephrine (µg/day)	1.003 (1.000–1.006)	0.049	**1.003 (1.000–1.007)**	**0.026**
Urine norepinephrine (µg/day)	1.001 (1.000–1.002)	0.004		
Preoperative highest HR (/min)	1.035 (1.011–1.061)	0.005	**1.046 (1.014–1.080)**	**0.005**

Data are shown as odds ratios (ORs) (95% confidence intervals).

## Discussion

The cornerstone treatment of PPGL is surgical resection. However, the surgery itself is accompanied by intraoperative HI and postoperative hypotension, which can lead to life-threatening complications. In the present study, intraoperative HI was present in 69.3% of subjects, and postoperative hypotension was present in 31.6% of PPGL patients after preoperative medical preparation. We demonstrated that the duration of α-adrenergic receptor blocker use was the only predictive factor for intraoperative HI and that a higher urine epinephrine level and greater preoperative highest HR were predictive factors for postoperative hypotension.

Previous researchers have focused on intraoperative HI and the related risk factors to improve the surgical outcome and reduce perioperative morbidity/mortality^11,12^. The definition of intraoperative HI was heterogeneous according to the studies, and there was no agreement regarding the definition of HI. Intraoperative hypertension may be related to intubation for general anesthesia, pneumoperitoneum during laparoscopic surgery, and PPGL manipulation. Intraoperative hypertension can lead to subsequent hypertensive crisis, consisting of various cardiac arrhythmias, left ventricular failure, myocardial ischemia, and stroke^2,3^. On the other hand, after surgical resection of the tumor, a sudden decline in catecholamine excess and the downregulation of adrenergic receptors due to catecholamine excess result in intraoperative hypotension. Since intraoperative hypotension is also related to postoperative complications, including acute kidney injury, myocardial injury^13^, stroke^14^, and even death^15^ we combined intraoperative hypertension and hypotension under the intraoperative HI definition. Therefore, we reported that the incidence of HI was 69.3% (n = 79), and only 8.8% (n = 10) of the subjects exhibited intraoperative hypertension. In our center, HI was caused mostly by intraoperative hypotension.

Median absolute performance error (MDAPE), average real variability (ARV), and hemodynamic instability score (HIS) were used to evaluate HI in the different operation fields. MDAPE was calculated through preoperative and postoperative SBP in noncardiac surgery, ARV was calculated though BP, and the HIS was calculated through hemodynamic variables, including BP, HR, volume therapy, and cardiovascular medication in high-risk abdominal surgery. In the present study, we found that there was a significant association of HI, MDAPE, and HIS.

Although some researchers have suggested that α-adrenergic receptor blockers might be harmful, causing orthostatic hypotension and intra- or postoperative hypotension^16–18^. preoperative use of α-adrenergic receptor blockers has been advocated by several guidelines^2,4,7^. Among several α-adrenergic receptor blockers, phenoxybenzamine was preferred. Phenoxybenzamine is a nonselective and noncompetitive α1- and α2-adrenergic receptor blocker with irreversible and long-duration effects. Thus, phenoxybenzamine is known to be superior to prevent intraoperative hypertension, but prolonged hypotension after surgery and reflex tachycardia are other concerns^19^. Selective and competitive α1-adrenergic receptor blockers, such as doxazosin, have a shorter duration effect and less associated reflex tachycardia^19^. Previous retrospective studies have yielded conflicting results^20–23^. The recent first randomized controlled trial comparing the efficacies of phenoxybenzamine and doxazosin demonstrated that the total duration of BP during surgery outside a predefined target range was not different between the two drugs^24^. Instead, the HIS was lower in the phenoxybenzamine group than in the doxazosin group, which was not linked with the cardiovascular complication rate^24^. However, we failed to show a significant difference between phenoxybenzamine users and nonusers in terms of intraoperative hypertension, postoperative hypotension, and even the HIS.

The present study demonstrated that the duration of α-adrenergic receptor blocker use was the only predictive factor for intraoperative HI. Tian et al.^25^ showed that the use of phenoxybenzamine for more than two weeks after the final dose adjustment could not further reduce the HI rate. In addition, Kong et al.^26^ demonstrated that the use of doxazosin for more than 30 days after the final dose adjustment might increase the risk of intraoperative bradycardia and postoperative hypotension. In the present study, duration of preoperative α-adrenergic receptor blocker use was defined as period from date of beginning of α-blockade to ending of α-blockade. Data collected from the electronic medical records, long duration of α-blockade was not associated with the difficulty to obtain target BP below 130/80 mmHg in supine position. Some patients with the long duration of preoperative α-blockade had been prescribed before diagnosis of PPGLs due to benign prostate hyperplasia or voiding problem. Without uncontrolled HI with appropriate medications for PPGL, elective operation was performed. Nine patients did not use α-adrenergic receptor blockers due to low BP or side effects, and 12 patients were prescribed less than 14 days. However, we cannot figure out the significant difference of incidence of HI between patients with a duration of α-blocker use ≥ 14 days and < 14 days. Patients who used α-blocker use ≥ 14 days were present in 66/79 (83.5%) without HI, and 28/35 (80.0%) with HI (P = 0.848). As the other authors acknowledged (Tian et al.^25^, Kong et al.^26^), the reason why the prolonged use of α-blocker is harmful to HI remained to be elucidated. But presumably, the prolonged use of α-blocker may suppress the vascular tone response to a sudden decrease of catecholamine after resection of PPGLs. However, to date, there was no clear evidence to support this. In our cohort, most patients (79 patients, 96.2%) with HI exhibited a MBP < 60 mmHg. Therefore, we suggest that prolonged use of alpha blocker was a predictor of only MBP < 60 mmHg. However, the underlying mechanism required further research.

Based on the results of the present study, we recognized the long-duration was not beneficial to prevent intraoperative HI, and needed further study to obtain the optimal duration of α-blockade for PPGL patients.

Contrary to previous data^9,10^, a larger tumor size was not a risk factor for HI in our study since the tumor size was relatively small compared with that in the other studies. In the present study, incidentally detected PPGLs during chest or abdominal computed tomography (CT) for the purpose of routine health check-ups were included.

There is controversy in previous studies regarding the effect of the surgical approach on HI. Kiernan et al.^10^ reported that subjects undergoing open surgery had a higher risk of HI than those undergoing laparoscopic surgery, but other studies reported that there was no increased risk of HI in open surgery groups^11,20^. Although open surgery was preferred in subjects with large PPGLs and the possibility of malignancy in our center, the surgical approach (open vs. laparoscopic) had no influence on HI.

The genetic testing results were available only in 63 of 114 patients, and only 10 of 63 patients harbored the genetic mutation: RET (n = 1), VHL (n = 4), SDHB (n = 1), SDHD (n = 3), and MAX (n = 1). Therefore, we could not analyze the HI between cluster 1 and cluster 2 tumors.

In the present study, postoperative hypotension occurred in 31.6% of subjects. Hypotension develops intraoperatively after tumor removal and may last for 24–48 h postoperatively, which requires fluid therapy and vasopressors. Premedication with phenoxybenzamine is known to be accompanied by a higher risk of postoperative hypotension compared to that with doxazosin premedication^22^. However, in our study, phenoxybenzamine was not a determinant for postoperative hypotension, in agreement with the PRESCRIPT study^24^. The high urine epinephrine level and elevated preoperative highest HR may reflect the extent of catecholamine secretion. Elevation in epinephrine levels downregulates β-adrenergic receptors in the heart, which depresses myocardial contractility^27^. After tumor removal during surgery, depressed myocardial function does not compensate for peripheral vasodilation, which might lead to intraoperative and postoperative hypotension^27,28^.

The major strength of the present study is detailed clinical and biochemical information, including the intraoperative and postoperative hemodynamic parameters, fluid management, and medication for PPGL patients undergoing surgical treatment. Intraoperative hemodynamic parameters were retrieved from the anesthetic records, which contain BP measurements at a 1-min resolution to evaluate HI. To evaluate postoperative hypotension, postoperative hemodynamic parameters were collected from electronic medical records obtained at the surgical intensive care unit for 24 h after surgery. We calculated the DDD of all antihypertensive medications. Thus, we could analyze the effect of the dosage of other antihypertensive medications as well as the use of α-adrenergic receptor blockers on HI.

This study has several limitations. First, this study was retrospective in a single institution and limited by selective and recall bias. Second, the sample size was relatively small, even though our institution is a large tertiary referral hospital. PPGLs are rare endocrine tumors with an overall prevalence of 2.13 per 100,000 in the Republic of Korea.

In conclusion, the present study revealed that intraoperative HI and postoperative hypotension were present in 69.3% and 31.6% of subjects who underwent surgery for PPGLs, respectively. Long-term use of α-adrenergic receptor blocker before surgery was significantly associated with intraoperative HI, and a higher urine epinephrine level and greater highest HR were predictive factors for postoperative hypotension. Therefore, we believe that the present study would be helpful to identify at-risk patients for intraoperative HI and postoperative hypotension and to guide delicate and intensive management in PPGL intraoperatively and postoperatively to improve clinical outcomes.

## Methods

### Study subjects

We recruited a total of 114 consecutive patients with PPGLs who underwent surgery at Seoul National University Hospital (Seoul, Korea) from March 2012 to April 2019. Patients who met the following criteria were excluded: (1) surgery due to carotid body tumor and (2) age less than 18 years. The present study was approved by the Institutional Review Board of the Seoul National University Hospital (No. H-1801–010-911), and performed in accordance with the Declaration of Helsinki. Informed consent was waivered because of the retrospective design in accordance with requirement of the Institutional Review Board of the Seoul National University Hospital.

### Preoperative variables

All clinical data were obtained from the medical records. Clinical information included i) age at initial diagnosis, sex, and body mass index; ii) comorbidities, such as diabetes mellitus, hypertension, coronary artery diseases, and cerebrovascular diseases, and history of abdominal surgery; iii) clinical symptoms/signs, including headache, sweating, palpitation, pain (neck, chest, abdomen, or bone), palpable mass, and hypertension (new onset, paroxysmal, or uncontrolled); iv) tumor characteristics, such as tumor size, side, metastasis, location, and serum/24-h urine catecholamine production, including epinephrine, norepinephrine, metanephrine, normetanephrine, and dopamine; v) preoperative hemodynamic parameters, including BP and HR before and after use of α-adrenergic receptor blockers; vi) preoperative medications, including α- and β-adrenergic receptor blockers and other antihypertensive drugs (antihypertensive medication regimens were calculated as the daily defined dose, DDD), which is the assumed average maintenance dose per day for a drug used for its main indication in adults, according to World Health Organization ATC/DDD Index 2019 (https://www.whocc.no/ddd)); and vii) American Society of Anesthesiologists physical status classification (ASA class).

PPGLs were diagnosed based on catecholamine excess before surgery and/or pathology after surgery. Catecholamine secretion was evaluated with 24-h urine catecholamine/fractionated metanephrines or serum fractionated metanephrines. The catecholamine type was classified as epinephrine, norepinephrine, or nonfunctioning type^29^. If urinary or serum epinephrine levels were elevated with or without high norepinephrine levels, the type was designated “epinephrine type”. If urinary or serum norepinephrine levels were elevated without high epinephrine levels regardless of dopamine levels, the type was classified as “norepinephrine type”. If both urinary or serum epinephrine and norepinephrine levels were within the normal range, the type was classified as “nonfunctioning type”. Serum fractionated metanephrines and urinary catecholamines were measured by liquid chromatography-tandem mass spectrometry (LC–MS/MS), and urinary fractionated metanephrines were assessed by high-performance liquid chromatography-electrochemical detection (HPLC-ECD).

Anatomical imaging, such as CT or magnetic resonance imaging (MRI), was used to locate PPGLs. Functional imaging, such as MIBG and positron emission tomography/computed tomography (PET/CT) with ^68^Ga-labeled DOTA-Tyr3 octreotide (DOTATOC), was performed to evaluate metastatic lesions. Metastasis was defined as the presence of pheochromocytomas in nonchromaffin organs, such as the lung, kidney, liver, bone, mediastinum, and lymph node, at diagnosis.

The ASA class was recorded from the patients’ electronic medical records.

### Preoperative medications

α-Adrenergic receptor blockers were administered to reach BP < 130/80 mmHg in the supine position and SBP > 90 mmHg in the upright position^4^. If several α-adrenergic receptor blockers were used, the final type and dose were included in the analysis. β-adrenergic receptor blockers were added in subjects with a HR at upright position > 100 bpm^4^. A high-salt diet and fluid intake were recommended. At our institution, we routinely performed preoperative infusion of saline (1–2 L) for several days (5–6 days) in all subjects except in cases with contraindication for volume expansion, to restore the catecholamine-induced volume depletion and prevent severe hypotension after tumor removal.

### Surgical techniques

Operations were performed under general anesthesia, and an arterial line was inserted into the patient’s radial artery after anesthesia induction for continuous arterial pressure monitoring. The surgical approach (open vs. laparoscopic) was determined based on the possibility of malignancy, tumor size, relationship to adjacent organs, and previous history of abdominal surgery. For most benign tumors < 7 cm on preoperative CT imaging, laparoscopic adrenalectomy was preferred.

### Definition of hemodynamic instability (HI)

HI was defined as one or more episodes of SBP > 200 mmHg or MBP < 60 mmHg during the surgery.

### Intraoperative variables

The hemodynamic variables, including BP and HR, were collected from the electronic anesthetic chart, which included vital signs with a 1-min interval during the operation. Fluid management, use of vasopressors and vasodilators, duration of surgery, and duration of anesthesia were also collected from electronic medical records.

We also analyzed HI using the HIS, ARV, and MDAPE. The HIS was calculated using the method reported by Buitenwerf et al^30^. The ARV and MDAPE were calculated using the following formulas^31–33^.

$$ARV = \frac{1}{N - 1}\mathop \sum \limits_{k = 1}^{N - 1} \left| {SBP_{k + 1} - SBP_{k} } \right|$$

$$MDAPE = median \left( {\left| {\frac{{SBP_{k} - SBP_{ward} }}{{SBP_{ward} }}} \right|} \right) \times 100\% ,$$

where SBP_ward_ represents the median ward BP measured one day before operation.

### Postoperative variables

After surgery, PPGL patients were routinely transferred to the surgical intensive care unit for close monitoring of hemodynamic status and moved to the ward on postoperative day 1. Postoperative clinical information included postoperative hemodynamic variables, fluid management, use of vasopressors and vasodilators, and postoperative complications, including postoperative mortality within 30 days.

Postoperative hemodynamic variables were collected from the electronic vital sign sheet in the intensive care unit for 24 h after surgery. Postoperative hypotension was defined as one or more episodes of MBP < 60 mmHg.

### Statistical analysis

All data are expressed as the mean ± SD, median (interquartile range) or absolute numbers (percentage) and were analyzed with SPSS software (version 25.0; IBM Corp., Armonk, NY, USA). In all analyses, P < 0.05 was considered to indicate statistical significance.

Continuous variables were compared with Student’s t-test or the Mann–Whitney U-test based on the results of the Shapiro–Wilk test. Categorical variables were compared using the Pearson chi-square test. Univariate logistic regression analysis was performed to identify predictive variables for intraoperative HI and postoperative hypotension. Multivariate logistic regression was performed using forward stepwise selection based on the Wald test. Cox proportional hazard ratio modeling for intraoperative HI was performed using the Python Lifelines library. Receiver operating characteristic (ROC) curve analysis was used to assess the prediction variables for intraoperative HI and postoperative hypotension.

## Supplementary Information

Supplementary Information 1.

Supplementary Information 2.

Supplementary Information 3.
