# Supplementary material for: Perioperative hemodynamic instability in pheochromocytoma and sympathetic paraganglioma patients
Source: Sci Rep. 2021 Sep 17;11:18574. doi: 10.1038/s41598-021-97964-3 (PMC8448751; doi:10.1038/s41598-021-97964-3)
Supplement: Supplementary file 1 — Supplementary Information 1. [file 41598_2021_97964_MOESM1_ESM.docx]

**Figure legends**

**Supplemental Figure 1.** Duration of α-blocker use according to hemodynamic instability (3 outliers are not shown).

The duration of α-adrenergic receptor blocker use was longer in subjects with HI than in subjects without HI (63.8 ± 57.3 vs. 40.7 ± 33.0 days, P=0.008).
