# Supplementary material for: Perioperative hemodynamic instability in pheochromocytoma and sympathetic paraganglioma patients
Source: Sci Rep. 2021 Sep 17;11:18574. doi: 10.1038/s41598-021-97964-3 (PMC8448751; doi:10.1038/s41598-021-97964-3)
Supplement: Supplementary file 2 — Supplementary Information 2. [file 41598_2021_97964_MOESM2_ESM.docx]

**Supplemental Table 1.** Intraoperative hemodynamic instability according to phenoxybenzamine use. Data are presented as the mean ± standard deviation or median (interquartile range).

|  | Phenoxy-  benzamine (-)  (n=89) | Phenoxy-  benzamine (+)  (n=25) | Total  (N=114) | *P*-value |
| --- | --- | --- | --- | --- |
| Hemodynamic instabilities | |  |  |  |
| - Time SBP > 200 (min) | 0.7 ± 3.0 | 0.2 ± 1.0 | 0.6 ± 2.7 | 0.339 |
| - Time MBP < 60 (min) | 12.6 ± 16.0 | 8.4 ± 13.7 | 11.7 ± 15.6 | 0.124 |
| - Area SBP > 200 (mmHg × min) | 20.6 ± 99.3 | 3.4 ± 17.2 | 16.8 ± 88.3 | 0.332 |
| - Area MBP < 60 (mmHg × min) | 72.6 ± 118.3 | 48.3 ± 76.5 | 67.3 ± 110.6 | 0.412 |
| - Std of SBP | 21.7 ± 5.8 | 21.1 ± 5.1 | 21.5 ± 5.6 | 0.641 |
| - HIS | 29.0 (21.0-40.0) | 22.0 (17.0-35.0) | 28.0 (19.2-36.8) | 0.120 |
| - ARV | 16.4 (12.3-20.0) | 16.0 (11.2-20.4) | 16.2 (12.1-20.0) | 0.803 |
| - MDAPE | 14.7 (12.3-19.2) | 14.0 (9.4-17.2) | 14.6 (11.5-18.9) | 0.239 |
| Fluid management |  |  |  |  |
| - Urine output (mL) | 240.0 (100.0-400.0) | 220.0 (100.0-450.0) | 240.0 (100.0-400.0) | 0.810 |
| - Estimated blood loss (mL) | 150.0 (0.0-400.0) | 50.0 (0.0-300.0) | 100.0 (0.0-350.0) | 0.212 |
| - Total infused fluid (mL) | 1450.0 (800.0-2000.0) | 1100.0 (700.0-1800.0) | 1375.0 (800.0-2000.0) | 0.166 |
| - Tidal transfused RBC (pack) | 0.0 (0.0-0.0) | 0.0 (0.0-0.0) | 0.0 (0.0-0.0) | 0.709 |
| Vasopressors |  |  |  |  |
| - Use of vasopressor | 0.8 ± 0.4 | 0.7 ± 0.5 | 0.8 ± 0.4 | 0.379 |
| - Number of vasopressors | 1.7 ± 1.2 | 1.6 ± 1.3 | 1.7 ± 1.2 | 0.642 |
| - Phenylephrine (µg) | 80.0 (0.0-270.0) | 40.0 (0.0-130.0) | 60.0 (0.0-200.0) | 0.169 |
| - Norepinephrine (µg) | 0.0 (0.0-42.2) | 0.0 (0.0-6.4) | 0.0 (0.0-37.4) | 0.437 |
| - Epinephrine (µg) | 0.0 (0.0-0.0) | 0.0 (0.0-0.0) | 0.0 (0.0-0.0) | 0.855 |
| - Ephedrine (mg) | 5.0 (0.0-20.0) | 0.0 (0.0-10.0) | 5.0 (0.0-15.0) | 0.149 |
| - Vasopressin (U) | 0.0 (0.0-0.0) | 0.0 (0.0-0.0) | 0.0 (0.0-0.0) | 0.354 |
| Vasodilators |  |  |  |  |
| - Use of vasodilator | 0.7 ± 0.5 | 0.8 ± 0.4 | 0.7 ± 0.5 | 0.320 |
| - Number of vasodilators | 1.2 ± 1.1 | 1.3 ± 0.9 | 1.2 ± 1.1 | 0.777 |

RBC, red blood cell; SBP, systolic blood pressure; MBP, mean blood pressure; Std, standard deviation; HIS, hemodynamic instability score; ARV, average real variability; MDAPE, median absolute performance error.
