# Supplementary figures and images for: Perioperative hemodynamic instability in pheochromocytoma and sympathetic paraganglioma patients
Source: Sci Rep. 2021 Sep 17;11:18574. doi: 10.1038/s41598-021-97964-3 (PMC8448751; doi:10.1038/s41598-021-97964-3)

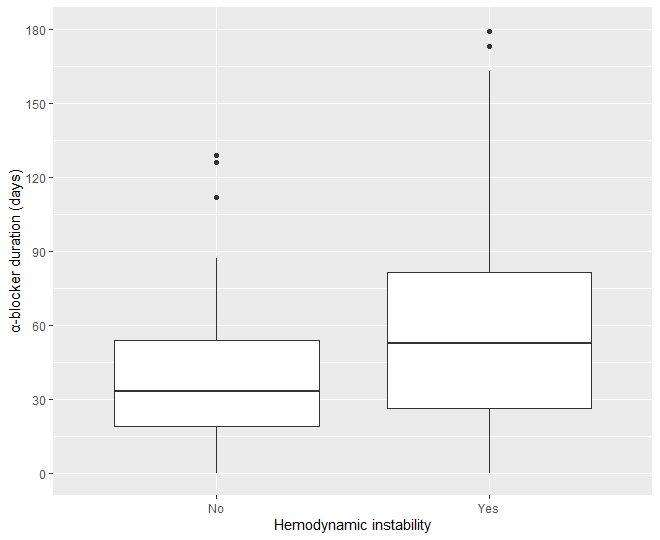

Supplement: Supplementary file 3 — Supplementary Information 3. [file 41598_2021_97964_MOESM3_ESM.tif]
